# Gut microbiota metabolites in the immunoregulation of enteritis: research progress

**DOI:** 10.3389/fimmu.2025.1706472

**Published:** 2025-12-08

**Authors:** Shaochen Yu, Mengjie Zhang, Ziyue Dou, Beibei Tian, Jian Lu

**Affiliations:** 1 Department of Emergency and Critical Care Medicine, Chuzhou Integrated Traditional Chinese and Western Medicine Hospital, Chuzhou, Anhui, China; 2 Department of Gastroenterology, The First Affiliated Hospital of Anhui Medical University, Hefei, Anhui, China

**Keywords:** gut microbiota metabolites, immunoregulation, inflammatory bowel disease, therapeutic strategies, intestinal barrier

## Abstract

The interaction between gut microbiota metabolites and the host immune system plays a crucial role in maintaining intestinal homeostasis and in the development of inflammatory bowel disease and other enteric conditions. This article presents a systematic review of the sources and functions of short-chain fatty acids, tryptophan metabolites, bile acids, and other microbial metabolites, focusing on how these metabolites regulate the function of immune cells, such as T cells, B cells, neutrophils, macrophages, and dendritic cells, as well as key inflammatory signaling pathways, including the NF-κB, NLRP3 inflammasome, and JAK–STAT pathways, thereby influencing intestinal barrier integrity. Also explored are potential therapeutic strategies based on microbial metabolites, including the application status and prospects of probiotic and prebiotic interventions, the direct administration of metabolites, and fecal microbiota transplantation. Although current research faces challenges such as unclear mechanisms, significant differences among individuals, and barriers to clinical translation, the development of multiomics technologies and precision medicine holds promise for providing more effective and personalized treatment strategies targeting gut microbiota metabolites for patients with enteritis.

## 1 Introduction

The composition and function of the gut microbiota interact intricately with the host immune system, a relationship that is vital for maintaining intestinal health. The gut microbiota not only participates in host nutritional metabolism but also regulates immune responses through its metabolites, thus playing a significant role in the onset and progression of enteritis (1). In recent years, research on the relationship between the gut microbiota and inflammatory bowel disease (IBD) has gradually increased, and findings have confirmed that gut dysbiosis is closely related to the pathogenesis of IBD (2, 3). Existing research indicates that an imbalance in gut microbial diversity, along with an overgrowth of specific pathogenic bacteria, can compromise intestinal barrier integrity, leading to chronic inflammation and immune dysregulation (4). As research has progressed, there has been a better understanding that gut dysbiosis not only causes local inflammation within the intestine but also may lead to systemic diseases by affecting the systemic immune system. For example, IBD patients often exhibit the overgrowth of certain specific microbes in their intestines. These microbes exacerbate inflammatory responses by producing proinflammatory metabolites and may cause extraintestinal immune dysregulation (4, 5). Focusing on the immunoregulatory mechanisms of these metabolites is pivotal. It clarifies the causal link between microbial dysbiosis and intestinal inflammation and pinpoints key molecular targets for novel therapies designed to reinstate immune homeostasis.

Research has shown that gut microbiota metabolites, such as short-chain fatty acids (SCFAs) and tryptophan metabolites, also play important roles in regulating immune responses and maintaining intestinal barrier integrity. For instance, SCFAs play key roles in regulating the function of intestinal epithelial cells and promoting the differentiation of regulatory T cells (Tregs), thereby suppressing inflammatory responses and maintaining intestinal immune homeostasis (6, 7). Tryptophan metabolites, such as indole-3-propionic acid (IPA), have been shown to enhance intestinal barrier function in inflammatory states and improve clinical outcomes (8). Therefore, understanding how the gut microbiota and its metabolites affect host immune responses is crucial for developing novel therapeutic strategies targeting the gut microbiota.

In recent years, treatment strategies based on gut microbiota products have attracted increasing attention. For example, fecal microbiota transplantation (FMT) has been used as a potential therapeutic method to alleviate IBD symptoms by restoring a healthy gut microbiota composition (9). Additionally, the use of probiotics and prebiotics has been shown to positively affect the gut microbiota, improving intestinal health and reducing inflammation levels (10).

In summary, the gut microbiota and its metabolites play key roles in the immunoregulation of enteritis. A deeper understanding of their mechanisms will provide new ideas and strategies for treating IBD and related diseases. These studies will both enhance our understanding of the interaction between the gut microbiota and host immunity and open new directions for future clinical treatments.

## 2 Gut microbiota and enteritis: an overview

A substantial body of evidence links dysbiosis of the gut microbiota to the pathogenesis of various forms of enteritis, including IBD, colitis, and infectious enteritis (11, 12). In IBD patients, a consistent reduction in microbial diversity and a shift in community structure, often characterized by a depletion of beneficial bacteria (e.g., *Faecalibacterium prausnitzii*) and an expansion of pro-inflammatory species (e.g., adherent-invasive *Escherichia coli*), are frequently observed (13). This imbalance disrupts host-microbe symbiosis, leading to compromised epithelial barrier function, aberrant activation of mucosal immune responses, and sustained inflammation (4, 5). The following sections will detail how metabolites derived from this dysbiotic microbiota serve as key mediators in the immunoregulation of enteritis.

## 3 Types and sources of gut microbiota products

### 3.1 Short-chain fatty acids

Short-chain fatty acids (SCFAs) are important metabolites produced by the fermentation of dietary fiber by the gut microbiota, with bacteria such as Bacteroidetes and Firmicutes playing key roles in this process (14). The main types of SCFAs include acetate, propionate, and butyrate. These SCFAs not only constitute the primary energy source for intestinal epithelial cells but also play important roles in maintaining intestinal barrier function and regulating immune responses and metabolism (15–17). Specifically, butyrate is considered among the most important SCFAs because it promotes the proliferation and differentiation of intestinal epithelial cells and has anti-inflammatory effects (18, 19). Importantly, SCFAs can regulate host energy metabolism and immune function by activating G protein-coupled receptors (GPCRs), which play significant roles in various physiological and pathological processes closely related to host health (20–22).

### 3.2 Tryptophan metabolites

Tryptophan is an important essential amino acid, and its metabolites play a crucial role in the interaction between the gut microbiota and the host. Tryptophan is primarily derived from microbiota such as *Lactobacillus* and *Bifidobacterium*; these microbes metabolize tryptophan to generate various bioactive metabolites, such as indole, indolepropionic acid, and kynurenine (23). These metabolites not only participate in regulating intestinal immune function (24) but also affect nervous system health (25), tumor development (26), and metabolic syndrome outcomes (27) and are closely related to mental health in some cases (28). One important metabolite, indole and its derivatives, can activate the aryl hydrocarbon receptor (AhR), exerting anti-inflammatory effects in regulating intestinal immune responses and barrier function (29, 30), further highlighting its importance in host health.

### 3.3 Bile acids and their derivatives

Bile acids are key metabolites synthesized by the liver and are transformed in the intestine by gut microbes. Primary bile acids synthesized in the liver are converted into secondary bile acids, such as deoxycholic acid and lithocholic acid, by intestinal microbial metabolism (31). These bile acids not only participate in fat digestion and absorption but also play important roles in regulating tumorigenesis, immunity, and microbial community composition (32–34). Bile acids can activate various nuclear receptors, such as the farnesoid X receptor (FXR) and Takeda G protein-coupled receptor 5 (TGR5), to regulate signaling molecules and the metabolism of carbohydrates, lipids, and energy, and play significant roles in modulating liver and intestinal metabolic functions (35, 36). Therefore, metabolic dysregulation may lead to various diseases, including nonalcoholic fatty liver disease and enteritis (37–39).

### 3.4 Other types of metabolites

In addition to short-chain fatty acids, tryptophan metabolites, and bile acids, the gut microbiota also produces various other metabolites that play important roles in intestinal health. Studies have shown that the gut microbiota can produce vitamins, antimicrobial peptides, and other bioactive compounds. These substances can not only enhance intestinal barrier function but also regulate immune responses and metabolic processes (39, 40). For example, the microbial decomposition product spermidine has anti-inflammatory effects and regulates physiological activities such as cell growth and proliferation (41, 42). Niechcial (43) et al. reported that spermidine has a protective effect on colitis and restores a healthy gut microbiota. Mechanistically, spermidine primarily maintains epithelial barrier integrity in a PTPN2-dependent manner and prevents macrophage polarization towards a proinflammatory phenotype to reduce intestinal inflammation. In addition to obtaining B vitamins from the diet, the gut microbiota is now considered a potential source of B vitamins (44). Studies have shown that B vitamins can, in turn, act as nutrients for the gut microbiota, regulators of immune cell activity, and modulators of colitis (45). Recently, Dalayeli (46) et al. validated the anti-inflammatory and antiulcer properties of B vitamins in animal models. They reported that B vitamins have a protective effect on mouse enteritis regardless of treatment dose and duration. These studies reveal the diversity and complexity of gut microbiota metabolites, emphasizing their importance in maintaining intestinal and systemic health.

## 4 Immunoregulatory mechanisms of gut microbiota metabolites

**Figure 1:**
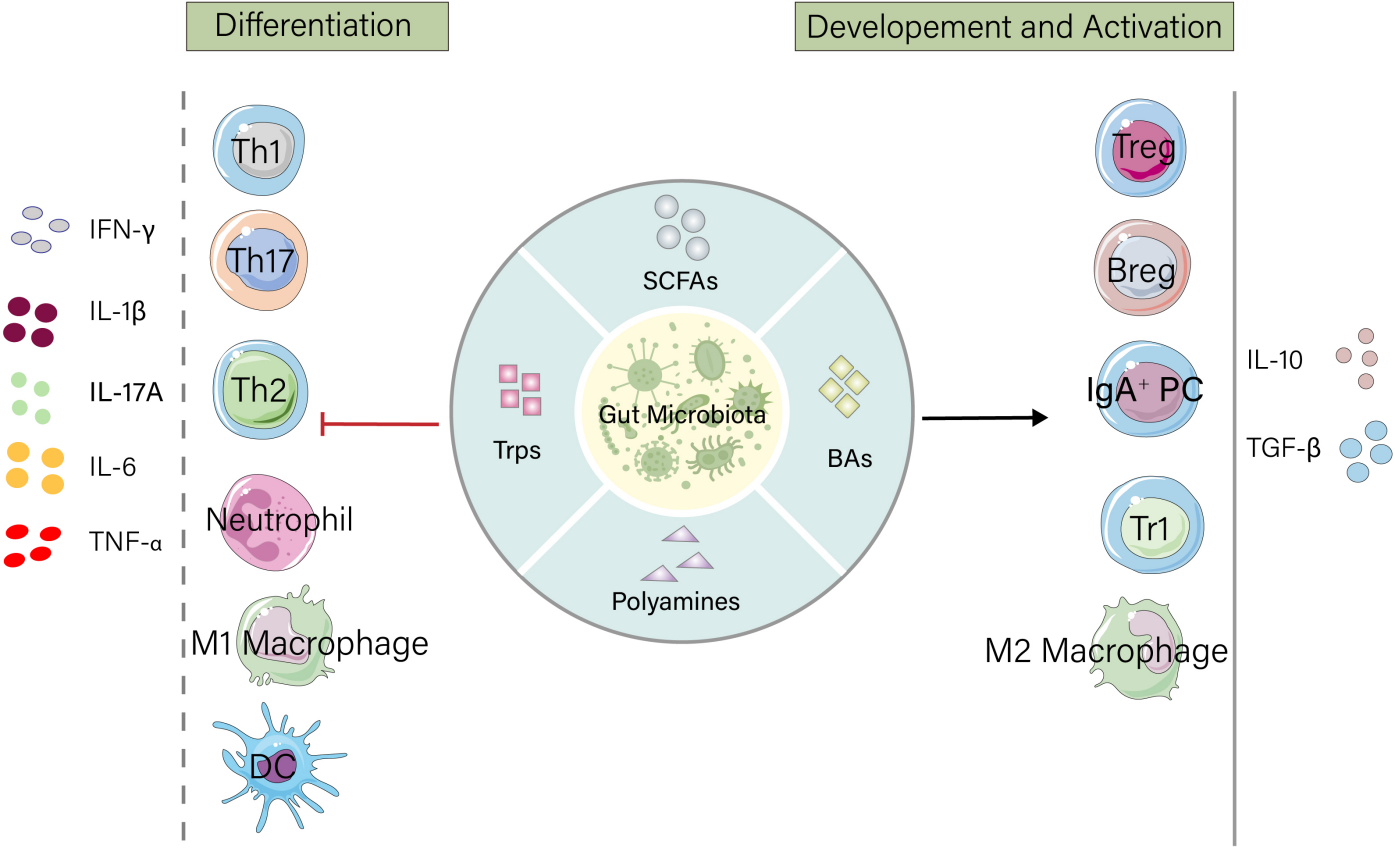
Gut microbiota metabolites maintain intestinal homeostasis by regulating the host immune system. Microbial products such as short-chain fatty acids (SCFAs), tryptophan, bile acids, and polyamines promote the development and activation of immunosuppressive cells, increase the release of anti-inflammatory factors, and suppress the differentiation of inflammatory cells as well as the secretion of proinflammatory factors. IFN, Interferon; IL, Interleukin; TNF, Tumor Necrosis Factor; Th, T helper cell; DC, Dendritic cell; Treg, Regulatory T cell; Breg, Regulatory B cell; IgA+PC, IgA-secreting plasma cell; Tr1, Type 1 regulatory T cell.

Gut microbiota metabolites exert broad and precise regulatory effects on the immune system. These metabolites not only directly influence the differentiation, activation, and function of various immune cells but also maintain intestinal immune homeostasis through mechanisms such as cellular metabolic reprogramming, epigenetic modifications, and signaling pathway activation. The following sections elaborate on the specific regulatory effects of gut microbiota metabolites on T cells, B cells, neutrophils, macrophages, and dendritic cells (**Figure 1**).

### 4.1 Modulation of adaptive immunity: T cells and B cells

T Cell Subset Differentiation: The gut microbiota precisely regulates the differentiation, function, and homeostasis of T cells through a network of metabolites, serving as a core mechanism for maintaining intestinal immune balance. SCFAs, produced by the symbiotic bacterial fermentation of dietary fiber, act as key signaling molecules. On the one hand, SCFAs perform epigenetic regulation by inhibiting histone deacetylases (HDACs) (16); on the other hand, they activate GPCRs (GPR43, GPR41, and GPR109a) (47). Together, these actions strongly promote the generation and activity of immunosuppressive Tregs while effectively inhibiting the overactivation of proinflammatory Th17 cells (47, 48) and regulating the metabolism and cytotoxicity of CD8^+^ T cells (49). Metabolites derived from the microbial metabolism of dietary tryptophan, such as indole derivatives and kynurenine, primarily activate the AhR signaling pathway, further inducing Treg differentiation, suppressing Th1/Th17 responses, and helping maintain intestinal epithelial barrier integrity and regulate T cell migration (50–52). Secondary bile acids, which are converted from primary bile acids, act as important signaling molecules by binding FXR or TGR5, also tending to inhibit the differentiation of proinflammatory Th17 cells, promote Treg generation (53, 54), and regulate T cell mitochondrial function and energy metabolism (55, 56). Furthermore, peptidoglycan fragments derived from bacterial cell wall degradation, particularly muramyl dipeptide (MDP), act as important pathogen-associated molecular patterns (PAMPs) recognized by the nucleotide-binding oligomerization domain-containing protein 2 (NOD2) receptor in host cells, and the activation of NOD2 signaling can inhibit the differentiation, expansion, and immunosuppressive function of Tregs (57). These diverse microbiota products (including substances such as polyamines and B vitamins that influence T cell metabolic reprogramming) work synergistically through three core pathways—epigenetic modification, specific receptor signal transduction, and cellular metabolic reprogramming—to finely maintain the dynamic balance between Tregs and effector T cells (such as Th1 and Th17 cells), thereby promoting immune tolerance and suppressing excessive inflammatory responses both locally and systemically (23, 58, 59). The disruption of this precise regulatory network dominated by microbiota products is closely related to the pathogenesis and development of various pathological states, such as IBD (60), autoimmune diseases (61), and cancer (62).

B Cell Development and Function: Gut microbiota metabolites precisely regulate the development, activation, differentiation, and function of B cells through multilayered direct and indirect mechanisms and play a central role in maintaining intestinal mucosal immune homeostasis. Like in T cells, SCFAs can also drive the differentiation of intestinal IgA^+^ plasma cells by activating GPCRs on the B cell surface and through epigenetic regulation via the inhibition of HDAC activity (63, 64). Moreover, SCFAs indirectly affect B cell affinity maturation and the germinal center (GC) responses by regulating the function of follicular helper T (Tfh) cells (65). Tryptophan metabolites (such as 5-hydroxyindole-3-acetic acid) enhance the suppressive function of Bregs and inhibit GC B cell and plasma cell differentiation by activating AhR in B cells (66). Additionally, polyamines (such as spermidine) regulate B cell metabolic adaptability and survival by inhibiting the mTORC1 pathway and enhancing autophagy (67). Furthermore, the gut microbiota can interact with vitamin A to maintain intestinal immune stability. With the assistance of the gut microbiota, the vitamin A derivative retinoic acid binds to the nuclear receptor RARα in B cells, induces the expression of the gut-homing receptor (α4β7 integrin), directs the recruitment of B cells to the intestinal mucosa, and promotes IgA secretion by B cells. IgA, in turn, can coordinately balance the composition of the gut microbiota (68). Overall, microbiota products achieve core regulation through direct receptor signaling (GPCRs/TLRs/AhR/RAR), epigenetic reprogramming, and indirect Tfh/Breg interaction networks, working synergistically to 1) induce immune tolerance and suppress inflammation via Bregs and 2) directionally recruit B cells to the intestine and optimize their response efficacy.

### 4.2 Influence on innate immunity: neutrophils, macrophages, and dendritic cells

Neutrophil Recruitment and Activation: Microbiota metabolites play a protective role in mucosal defense and immune homeostasis by regulating neutrophil recruitment, activation, bactericidal function, and inflammation resolution pathways. SCFAs induce neutrophil migration to inflammation sites and increase their phagocytic capacity by activating the cell surface GPR43 receptor (69), significantly reducing the release of neutrophil chemokines (such as IL-8/CXCL1) and proinflammatory cytokines (such as IFN-γ and IL-1β) (70) while decreasing the release of neutrophil reactive oxygen species (ROS) to mitigate tissue damage (71), thereby limiting the inflammatory response capacity of neutrophils. Tryptophan metabolites activate AhR, inhibiting NLRP3 inflammasome activation and IL-1β maturation (72, 73) while also inhibiting neutrophil myeloperoxidase (MPO) activity (74), accelerating the resolution of neutrophil-mediated inflammation and reducing bystander tissue damage. Specific microbiota metabolites, such as succinate, which is overproduced by pathogenic *Fusobacterium nucleatum*, can promote neutrophil extracellular trap (NET) formation. NETs further increase the proportion of Th1/Th17 cells among CD4^+^ T cells and the expression of the proinflammatory cytokines IFN-γ/IL-17A, thereby amplifying autoimmune inflammation (75, 76). In the context of cancer, bile acids induce neutrophil recruitment and maintain their immature phenotype via the GPBAR1-CXCL10 axis, creating an immunosuppressive tumor microenvironment (76). In summary, microbiota products achieve a bidirectional fine balance in regulating neutrophils: on the one hand, they can enhance phagocytic function to strengthen defense against acute infections; on the other hand, they can suppress excessive inflammatory factor release and promote inflammation resolution and tissue repair. An imbalance in this regulatory network can lead to abnormal neutrophil activation or functional defects, which are deeply involved in the pathological processes of diseases such as IBD, sepsis, and autoimmune diseases.

Macrophage Polarization and Function: Macrophages play important roles in immune responses, and their function is also regulated by gut microbiota products. Gut microbiota products continuously and precisely regulate the development, polarization, functional status, and tissue homeostasis maintenance of intestinal macrophages. These microbial small molecules act as key commensal signals on macrophages in different intestinal regions (such as the lamina propria, Peyer’s patches, and crypts) through various mechanisms: on one hand, they specifically bind to macrophage GPRs [e.g., GPR43 binds SCFAs (77), and TGR5 binds secondary bile acids (78)] or intracellular receptors [e.g., AhR binds tryptophan metabolites (79), and NOD2 recognizes muramyl dipeptide (80)], triggering downstream complex signaling networks [e.g., inhibiting the PI3K/Akt/mTOR and NF-κB proinflammatory pathways (81) and inducing cAMP/PKA signaling (82)] and profoundly affecting epigenetic modifications [e.g., among SCFAs, butyrate potently inhibits histone deacetylases (HDACs), increases histone acetylation levels, and opens chromatin for anti-inflammatory genes (83)]; on the other hand, through this multireceptor, multipathway integration, they drive macrophages towards an anti-inflammatory, reparative, and tolerant phenotype (M2-like), manifested as a significantly enhanced phagocytic clearance capacity (efficiently clearing apoptotic cells and bacterial debris), an increase in the secretion of key anti-inflammatory cytokines (such as IL-10, TGF-β), and the suppression of proinflammatory mediators like reactive oxygen species, while strongly inhibiting the excessive activation of macrophages towards a proinflammatory state (M1-like) and reducing the release of proinflammatory cytokines (such as TNF-α, IL-6) (84, 85). Furthermore, specific microbiota products (like butyrate) are crucial for maintaining the local self-renewal, survival, and homeostatic pool of embryo-derived macrophages within the intestinal lamina propria (83, 86), influence the differentiation of bone marrow-derived monocytes into macrophages (86), “train” macrophages to establish immune tolerance to commensal bacteria (avoiding unnecessary inflammatory responses) while retaining vigilance against pathogens (6, 87), and strengthen intestinal barrier integrity by promoting epithelial tight junction protein expression and goblet cell mucus secretion (86, 88). This multifaceted and dynamically balanced regulatory network dominated by microbiota metabolites is the cornerstone of intestinal immune homeostasis, effectively coordinating the host defence, damage repair, and harmonious coexistence with commensal bacteria.

Dendritic Cell Maturation and T Cell Priming: Dendritic cells (DCs) are important bridges connecting innate and adaptive immunity, and their function is also regulated by gut microbiota products. Gut microbiota metabolites are core environmental signals that shape the phenotype, function, and immune response of intestinal DCs; they regulate DC maturation, migration, cytokine secretion profiles, antigen presentation efficiency, and the ability to induce T cell differentiation through various molecular mechanisms, thus playing a pivotal role in maintaining intestinal homeostasis and immune tolerance. These microbe-derived molecules (mainly including SCFAs, tryptophan metabolites, secondary bile acids, etc.) are recognized by DC subsets in different intestinal regions (such as CD103^+^CD11b^+^, CD103^+^CD11b^-^, CD11b^+^CD103^-^, and plasmacytoid DCs) by binding their surface or intracellular specific receptors [GPR43 binds SCFAs (88), AhR binds tryptophan metabolites (89), and FXR/TGR5 binds bile acids (90)], triggering downstream key signaling pathways [e.g., inhibiting NF-κB proinflammatory signaling via TGR5-cAMP-PKA (91)] and influencing epigenetic modifications [(e.g., butyrate inhibiting HDAC activity (92)]. Under this influence, microbiota products strongly drive DCs to present a “semimature” or “regulatory” phenotype, characterized by the moderate upregulation of the expression of costimulatory molecules (such as CD80, CD86, and CD40) and major histocompatibility complex class II molecules (MHC-II) to maintain necessary antigen presentation capacity (93), while selectively enhancing the secretion of anti-inflammatory/regulatory cytokines (such as IL-10 and TGF-β) and immunoregulatory mediators (e.g., retinoic acid metabolizing enzymes, e.g., RALDHs, catalyzing retinoic acid production and indoleamine 2,3-dioxygenase activating IDO1) (59, 94). This unique cytokine and mediator environment prompts DCs to preferentially induce the differentiation of naïve T cells into Foxp3^+^ Tregs and IL-10-producing type 1 regulatory T cells (Tr1) rather than proinflammatory Th1, Th2, or Th17 cells, thereby actively establishing and maintaining immune tolerance to food antigens and commensal bacteria (95).

## 5 Regulation of inflammatory signaling pathways by gut microbiota products

**Figure 2:**
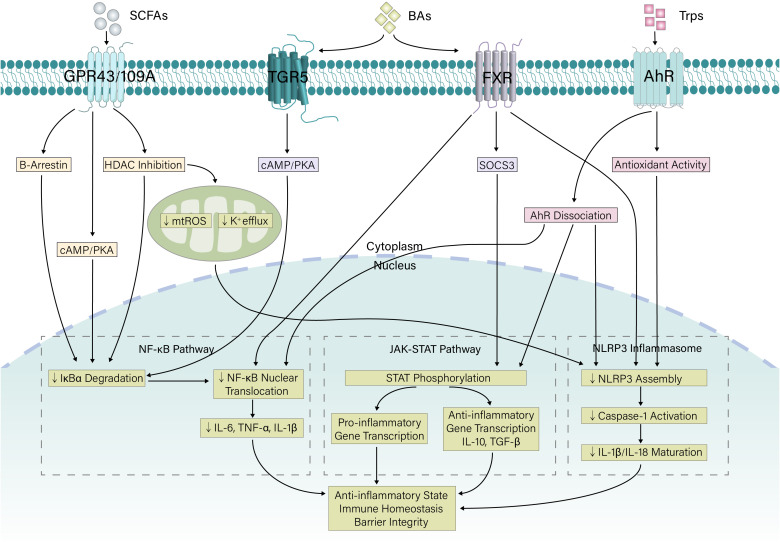
Coordinated regulation of inflammatory signaling by microbial metabolites. Gut microbiota-derived metabolites (SCFAs, bile acids, and tryptophan metabolites) signal through specific cellular receptors to suppress proinflammatory pathways. SCFAs act via GPCRs (GPR43/109A) and HDAC inhibition to stabilize IκBα and improve mitochondrial function. Bile acids signal through TGR5 and FXR to inhibit NF-κB and induce SOCS3. Tryptophan metabolites activate AhR and exert antioxidant effects. Together, these compounds synergistically inhibit NF-κB activation and NLRP3 inflammasome assembly and modulate JAK–STAT signaling, promoting an overall anti-inflammatory state and immune homeostasis. SCFA, short-chain fatty acid; Trp, Tryptophan; GPCR, G-protein coupled receptor; FXR, Farnesoid X receptor; AhR, Aryl hydrocarbon receptor; HDAC, histone deacetylase; SOCS3, suppressor of cytokine signaling 3; mtROS, mitochondrial reactive oxygen species; cAMP, Cyclic Adenosine Monophosphate; PKA, Protein Kinase A; TGF, Transforming Growth Factor; IL, Interleukin; TNF, Tumor Necrosis Factor.

In addition to directly regulating immune cells, gut microbiota metabolites also serve as key modulators of intracellular inflammatory signaling pathways. By activating or inhibiting specific receptors and kinases, gut microbiota metabolites precisely regulate the activity of downstream transcription factors and the expression of inflammation-related genes, playing a decisive role in maintaining intestinal immune balance. The following sections elaborate on the regulatory effects of major metabolites on critical signaling pathways, including the NF-κB, NLRP3 inflammasome, and JAK–STAT pathways (**Figure 2**).

### 5.1 NF-κB signaling pathway

The gut microbiota finely and complexly regulates the host NF-κB pathway through its metabolites, a process that is crucial for maintaining intestinal immune homeostasis. The core mechanisms include the following: short-chain fatty acids (e.g., butyrate) stabilize IκBα by inhibiting HDAC (96) and activate GPR43/GPR41 to trigger β-arrestin-2 or cAMP/PKA signaling, collectively blocking NF-κB nuclear translocation and transcriptional activity (97, 98); tryptophan activates AhR, which directly antagonizes the NF-κB subunit p65 in the nucleus, inhibiting proinflammatory gene expression (99); bile acids inhibit IKK by activating the membrane receptor TGR5 (100), while nuclear receptor FXR activation induces the inhibitory protein SHP, dually inhibiting NF-κB activation (101); and microbiota metabolites indirectly inhibit the excessive activation of the TLR/NF-κB pathway by maintaining epithelial barrier integrity and reducing exposure to pathogen-associated molecular patterns (such as LPS) (102). Once this dynamic balance is disrupted (dysbiosis), harmful bacterial products (such as LPS) drive strong TLR4-mediated IKK phosphorylation, leading to sustained NF-κB activation and the development of chronic inflammatory diseases (such as IBD and metabolic syndrome) (103, 104). Therefore, by directly intervening in kinase activity and nuclear receptor interactions, the microbiota metabolic network precisely regulates the intensity of NF-κB signaling, serving as a core hub for intestinal immune balance.

### 5.2 The NLRP3 inflammasome

Gut microbiota metabolites precisely regulate NLRP3 inflammasome activation through bidirectional, dynamic mechanisms, processes that are crucial for maintaining intestinal immune balance. On the one hand, specific microbiota products can directly activate or sensitize NLRP3: extracellular ATP released by bacterial death triggers K^+^ efflux and Ca²^+^ influx via the P2X7 receptor; pore-forming toxins (e.g., α-toxin) produced by pathogens disrupt cell membrane ion homeostasis; and bacterial nucleic acids/particles upregulate NLRP3 and pro-IL-1β expression via TLRs, initiating NF-κB signaling, collectively driving inflammasome assembly and IL-1β/IL-18 maturation and release (105). On the other hand, beneficial metabolites from commensal bacteria constitute a powerful inhibitory network: short-chain fatty acids (e.g., butyrate) directly block NLRP3 oligomerization and ASC speck formation by inhibiting HDAC (106) and activate the GPR43 receptor, inducing β-arrestin-2 to inhibit inflammatory pathways (97), and tryptophan activates AhR, enhances mitophagy to clear damaged mitochondria, upregulates the expression of antioxidant genes, and directly inhibits NLRP3 transcription (107).

### 5.3 JAK–STAT signaling pathway

Gut microbiota metabolites precisely regulate the JAK–STAT signaling pathway in host cells through multilevel mechanisms, playing a core role in maintaining immune homeostasis and inflammatory balance. The core regulatory strategies include the following: 1) directly inhibiting kinase and STAT activation: SCFAs (e.g., butyrate) upregulate the expression of the negative regulatory protein SOCS3 by inhibiting HDAC activity and blocking JAK kinase activity and STAT (e.g., STAT3/STAT5) phosphorylation (108); simultaneously activating GPR43/GPR41 interferes with the STAT cascade (109); and tryptophan metabolites (indole derivatives, etc.) promote STAT degradation through AhR–STAT protein interactions (110); 2) Nuclear receptor-mediated transcriptional regulation: Bile acids activate the nuclear receptor FXR, downregulate the expression of cytokine receptors such as IL-6R and induce SOCS3, indirectly inhibiting the IL-6-JAK–STAT pathway (111).

## 6 Regulation of intestinal barrier function by gut microbiota products

### 6.1 Enhancing tight junction protein expression

Various bioactive molecules produced by gut microbiota metabolism increase the expression and functional integrity of tight junction proteins between epithelial cells through complex and synergistic mechanisms, thereby consolidating the mucosal barrier. Among such molecules, SCFAs (e.g., butyrate and propionate) play a central role: on the one hand, as histone deacetylase inhibitors (HDACis), they directly promote the transcriptional activation of core tight junction proteins (e.g., occludin, claudins, and ZO-1) by increasing histone acetylation levels in the promoter regions of target genes (112, 113); on the other hand, SCFAs activate GPRs (e.g., GPR41, GPR43, and GPR109a) on the surface of intestinal epithelial cells, triggering intracellular signaling cascades, such as activating the AMPK/mTOR pathway, thereby regulating the synthesis, phosphorylation status and localization of tight junction proteins to the cell membrane, thus enhancing junction stability (114, 115). Additionally, tryptophan metabolites (e.g., indole and its derivatives) act as AhR ligands, activating the AhR signaling pathway and inducing downstream target gene expression, including directly or indirectly promoting the transcription of various tight junction proteins and inhibiting the production of proinflammatory factors, thus reducing inflammation-induced damage to tight junctions (116, 117). Secondary bile acids regulate the expression of tight junction-related genes by activating FXR or the membrane receptor TGR5 (118, 119). Moreover, some polyamines produced by bacteria can promote cell proliferation and repair, indirectly supporting the maintenance of tight junction structures (120). These microbial products also generally have potent anti-inflammatory effects, reducing the levels of cytokines such as TNF-α and IFN-γ by inhibiting proinflammatory pathways such as the NF-κB pathway. These inflammatory factors are known to be potent inhibitors and disruptors of tight junction protein expression (121, 122). Therefore, gut microbiota metabolites work synergistically through multiple targets (epigenetic regulation, receptor signaling, and anti-inflammatory activity) to increase the expression abundance and function of tight junction proteins, establishing and strengthening the physical defence against external harmful substances, serving as an indispensable driving force for maintaining intestinal epithelial barrier homeostasis.

### 6.2 Regulating intestinal epithelial cell proliferation and apoptosis

Studies have shown that SCFAs promote the proliferation of intestinal epithelial cells by activating the Wnt/β-catenin signaling pathway (123, 124). This process is crucial for maintaining the integrity and function of the intestinal epithelium. In mouse models, SCFA supplementation has been shown to increase the proliferative capacity of intestinal epithelial cells and promote cell growth and regeneration, thereby accelerating repair after injury (125). Furthermore, SCFAs promote intestinal epithelial proliferation and repair by regulating the expression of cell cycle-related proteins (such as Cyclin D3), which has potential clinical significance for treating intestinal inflammation (126).

The metabolism of secondary bile acids (e.g., deoxycholic acid) in the intestine has also been shown to play important roles in regulating the survival and apoptosis of intestinal epithelial cells. Studies indicate that secondary bile acids can inhibit the necroptosis of intestinal epithelial cells by activating FXR, thereby enhancing intestinal barrier function (127). In mouse DSS-induced colitis models, treatment with secondary bile acids significantly reduced the apoptosis rate of intestinal epithelial cells, promoted epithelial cell proliferation, and improved the overall health status of the intestine (128). Additionally, the regulatory effect of secondary bile acids on the gut microbiota also supports the mechanism by which they protect the intestinal epithelium by altering the composition of gut microbes, further enhancing intestinal barrier integrity and function (31).

## 7 Potential therapeutic strategies based on gut microbiota products

### 7.1 Probiotic and prebiotic interventions

Based on the core mechanism through which gut microbiota metabolites regulate intestinal homeostasis, probiotic and prebiotic interventions have become promising strategies for treating enteritis (such as IBD). The core strategy is to restore homeostatic levels of microbiota products with barrier repair and anti-inflammatory effects by reshaping the dysregulated microbial community and its metabolic activity, thereby alleviating intestinal inflammation (129). Probiotics (e.g., specific strains of Bifidobacterium and Lactobacillus) directly supplement symbiotic bacteria that produce SCFAs, tryptophan metabolites, or secondary bile acids; while colonizing or transiently surviving in the gut, they not only competitively inhibit pathogen growth but also directly secrete metabolites such as SCFAs, indole compounds, and polyamines (130–133). These substances synergistically repair the physical barrier and suppress local inflammatory responses by promoting epithelial repair or enhancing tight junction protein expression. Moreover, prebiotics (such as pectin, resistant starch, fructooligosaccharides, and other indigestible fibers) act as selective substrates, specifically stimulating the expansion and metabolism of host SCFA-producing bacteria (such as *Eubacterium* and *Roseburia*), significantly increasing intestinal concentrations of SCFAs and markedly increasing the overall richness of the microbiota (131). Furthermore, the combined use of probiotics and prebiotics (synbiotics) can produce synergistic effects, and the functional efficacy of synbiotics is superior to that of probiotics or prebiotics alone (134), demonstrating good potential value for intestinal health interventions. Clinical evidence has shown that synbiotics as adjuvant therapy can alleviate symptoms and are well tolerated by patients with mild to moderate ulcerative colitis (UC), but the differences in efficacy among different formulations still need to be investigated further (135, 136). Notably, the effects of probiotics in the remission phase of UC are relatively limited (137), and there are application limitations such as low bioactivity and short retention times (138, 139), prompting researchers to explore alternative strategies such as postbiotics (140). Novel prebiotics such as functional oligosaccharides show therapeutic potential by regulating microbiota metabolic pathways (141), but more rigorously designed clinical trials are needed to verify their specific efficacy (142). Overall, such microecological modulators, as multitarget intervention strategies, have important application prospects in the comprehensive management of UC (143).

### 7.2 Direct administration of microbiota metabolites

UC patients often lack certain microbiota metabolites, and these molecules play key roles in maintaining intestinal homeostasis (31, 144, 145). In preclinical studies, direct supplementation with these products has shown encouraging results. For example, in DSS- or TNBS-induced mouse colitis models, the administration of sodium butyrate via enema or drinking water not only significantly improved the disease activity index, reduced histological damage, and decreased proinflammatory cytokine levels but also promoted the restoration of the gut microbial community balance (146); similarly, the administration of the ellagic acid metabolite urolithin A also effectively alleviated inflammation in animal models (147). Although clinical translation is not yet a reality, preliminary evidence from human trials supports the potential of the direct administration of microbiota metabolites. For instance, several small clinical trials have shown that oral butyrate as an adjunctive therapy for UC can alleviate clinical symptoms, improve endoscopic scores, and reduce histological inflammation, with good safety (148–150). Indole derivatives (such as IPA and indole-3-aldehyde) produced by the microbial metabolism of tryptophan are key ligands for AhR. The activation of AhR signaling is crucial for maintaining epithelial barrier integrity, promoting IL-22-mediated repair, and regulating immune cell function (151). Furumatsu (152) et al. reported that mice lacking AhR are more susceptible to colitis than wild-type mice are. Providing these ligands (e.g., oral indole-3-carbinol) as a supplement in mouse colitis models significantly alleviated colitis (153).

The direct administration of gut microbiota products represents a “functional replacement” precision treatment strategy. This strategy aims to repair damaged intestinal homeostasis by supplementing key ecological niche molecules missing in UC patients and directly acting on host epithelial and immune cells, opening a highly promising new direction for developing the next generation of efficient and safe UC therapies.

### 7.3 Fecal microbiota transplantation

Fecal microbiota transplantation (FMT), as an emerging treatment method, has progressed from initial validation to in-depth mechanistic exploration and protocol optimization in the latest research on intestinal disease treatment. The current consensus is that FMT shows significant and promising efficacy for refractory UC (154). Multiple randomized controlled trials (RCTs) have indicated that the rate of clinical remission induced by multiple FMT procedures via colonoscopy can exceed 50%. The core mechanisms of FMT include reconstructing the intestinal microecological balance, restoring the function of beneficial microbes (such as SCFA-producing bacteria), strengthening intestinal barrier integrity, and regulating host immune responses (e.g., increasing Tregs) (155–158). However, efficacy is highly dependent on the donor, and the “superdonor” phenomenon (i.e., the microbiota from certain healthy donors consistently yields better efficacy) has become a research focus; the microbiota characteristics of superdonors typically include high diversity and specific metabolic functions (156, 157). In contrast, evidence for FMT in patients with Crohn’s disease (CD) remains weak and inconsistent, but FMT has shown potential for alleviating concurrent *Clostridium difficile* infection and some intestinal symptoms (159).

## 8 Limitations and future directions

Although significant progress has been made in recent years regarding the role of the gut microbiota and its metabolites in the immunoregulation of enteritis, the current research still has certain limitations. First, most studies are still in the basic research stage, clinical trials are relatively limited, and sample sizes are small, making it difficult to generalize results to broader populations (160). Second, the high individual variability in the gut microbiota leads to considerable fluctuations in the effectiveness of interventions, and unified standardized treatment protocols are lacking (161). Furthermore, the interactions between different microbiota metabolites (such as SCFAs, tryptophan metabolites, and bile acids) and their specific regulatory mechanisms in the complex *in vivo* environment are not fully understood (162), especially their dynamic changes under different pathological conditions, which still require further exploration (163, 164).

The following research directions should be considered in future studies: First, in-depth analyses of the specific mechanisms of action of particular microbiota metabolites on immune cell signaling pathways, especially their differential effects on different cell types and inflammatory environments (162, 165), are warranted; second, more high-quality, large-sample clinical studies should be conducted to validate the efficacy and safety of strategies such as probiotics, prebiotics, postbiotics, and the direct administration of microbiota metabolites (160, 166); third, multiomics technologies (such as metagenomics, metabolomics, and single-cell sequencing) should be combined to establish personalized gut microbiota intervention models for precision treatment (161); and fourth, the extended effects of microbiota–host interactions in extraintestinal organs (such as lungs and joints) and systemic diseases should be explored, expanding the clinical application scope of gut microbiota regulation (167, 168).

## 9 Conclusion

In summary, the gut microbiota and its metabolites play crucial roles in the immunoregulation of enteritis. Through multiple mechanisms, such as regulating immune cell function, influencing inflammatory signaling pathways, and enhancing intestinal barrier integrity, these processes collectively maintain intestinal homeostasis. Current research not only reveals the close relationships among microbiota diversity, metabolic functions, and the pathogenesis and progression of enteritis but also provides a theoretical basis for the development of novel microbiota-based treatment strategies (such as probiotics and FMT).

Although challenges such as unclear mechanisms, significant individual differences, and difficulties in clinical translation remain (160), with the continuous advancement of research methods and in-depth interdisciplinary collaboration, the application prospects of gut microbiota products in enteritis treatment could be very broad. Future studies should continue to explore the underlying mechanisms in greater detail, optimize intervention strategies, and promote clinical translation. Ultimately, this work will provide more effective and personalized treatment plans for patients with enteritis, improving their quality of life and health outcomes.
